# Bacteriophages vB_Sen-TO17 and vB_Sen-E22, Newly Isolated Viruses from Chicken Feces, Specific for Several *Salmonella enterica* Strains

**DOI:** 10.3390/ijms21228821

**Published:** 2020-11-21

**Authors:** Katarzyna Kosznik-Kwaśnicka, Łukasz Grabowski, Michał Grabski, Mateusz Kaszubski, Marcin Górniak, Agata Jurczak-Kurek, Grzegorz Węgrzyn, Alicja Węgrzyn

**Affiliations:** 1 Laboratory of Molecular Biology, Institute of Biochemistry and Biophysics, Polish Academy of Sciences, Kładki 24, 80-822 Gdansk, Poland; k.kwasnicka@hotmail.com (K.K.-K.); lukas.grabowski95@gmail.com (Ł.G.); 2 Department of Molecular Biology, University of Gdansk, Wita Stwosza 59, 80-308 Gdansk, Poland; michal.grabski@phdstud.ug.edu.pl (M.G.); mateuszkaszubski5@wp.pl (M.K.); grzegorz.wegrzyn@biol.ug.edu.pl (G.W.); 3 Institute of Oceanology, Polish Academy of Sciences, Powstańców Warszawy 55, 81-712 Sopot, Poland; 4 Department of Molecular Evolution, University of Gdansk, Wita Stwosza 59, 80-308 Gdansk, Poland; marcin.gorniak@ug.edu.pl (M.G.); agata.jurczak-kurek@ug.edu.pl (A.J.-K.)

**Keywords:** bacteriophages, *Salmonella*, lytic development, genomic analysis

## Abstract

Two newly discovered bacteriophages, isolated from chicken feces and infecting *Salmonella enterica* strains, are described in this report. These phages have been named vB_Sen-TO17 and vB_Sen-E22, and we present their molecular and functional characterization. Both studied viruses are able to infect several *S. enterica* strains and develop lytically, but their specific host ranges differ significantly. Electron microscopic analyses of virions have been performed, and full genome sequences were determined and characterized, along with molecular phylogenetic studies. Genomes of vB_Sen-TO17 (ds DNA of 41,658 bp) and vB_Sen-E22 (dsDNA of 108,987 bp) are devoid of homologs of any known or putative gene coding for toxins or any other proteins potentially deleterious for eukaryotic cells. Both phages adsorbed efficiently (>95% adsorbed virions) within 10 min at 42 °C (resembling chicken body temperature) on cells of most tested host strains. Kinetics of lytic development of vB_Sen-TO17 and vB_Sen-E22, determined in one-step growth experiments, indicated that development is complete within 30–40 min at 42 °C, whereas burst sizes vary from 9 to 79 progeny phages per cell for vB_Sen-TO17 and from 18 to 64 for vB_Sen-E22, depending on the host strain. Virions of both phages were relatively stable (from several percent to almost 100% survivability) under various conditions, including acidic and alkaline pH values (from 3 to 12), temperatures from −80 °C to 60 °C, 70% ethanol, chloroform, and 10% DMSO. These characteristics of vB_Sen-TO17 and vB_Sen-E22 indicate that these phages might be considered in further studies on phage therapy, particularly in attempts to eliminate *S. enterica* from chicken intestine.

## 1. Introduction

Among various foodborne pathogenic bacteria, *Salmonella enterica* is one of the major infection agents responsible for human diseases caused by contamination of poultry-derived products [1]. While this bacterium usually does not induce any disorders in birds, it can be dangerous to humans. Abundance of *S. enterica* in chicken gut may result in contamination of poultry-derived food appearing during the production process [2]. There are various serotypes of *S. enterica* which can cause diseases in humans, and the problem of salmonellosis is global [3]. In many countries, detection of specific serotypes of *S. enterica* (in most cases Typhimurium and Enteritidis) in poultry is considered as a compulsion to liquidate the whole flock [1].

Eradication of *S. enterica* can be performed using antibiotics; however, this method has met serious and global problems [4]. Bacterial strains resistant to many antibiotics are already known, which causes real crisis in therapies of infectious diseases [5,6]. Therefore, it is clear that development of novel methods of treatment of diseases caused by bacteria is necessary [7,8]. Phage therapy, i.e., the use of bacteriophages to combat bacterial infections, is one of the possible options [9]. Nevertheless, development of this method is not simple and requires specific conditions [9,10,11]. It is necessary to establish collections of potentially useful phages which can kill bacteria by conducting lytic development. Since phages are usually very specific to their hosts, such collections should be large to give a possibility of finding viruses capable of propagation in a particular bacterial strain isolated from patients or infected animals. Such bacteriophages must not contain genes coding for toxins and other proteins that might cause damage of human or animal cells. Therefore, it is still necessary to isolate previously unknown bacteriophages and to characterize them [9,10,11].

The proposal of the use of phage therapy to treat *Salmonella* infections has been published previously in many reports. Those studies led to determination of properties of various *Salmonella*-specific phages [12,13,14,15,16,17,18,19,20,21], effects of application of phages to poultry [22,23,24,25,26,27], and results of experimental use of phages in therapies of infected animals [28,29,30,31,32]. In addition, economic analyses of potential use of anti-*Salmonella* phage therapy in poultry have also been published [1,33]. Despite promising results of these studies, it was also indicated that most phages infecting *S. enterica* have a relatively narrow host range, specific to one or a few serovars or strains [12,13,14,15,16,17,18,19,20,21,34]. Thus, characterization of newly isolated bacteriophages infecting this bacterium is reasonable. Following this line of studies, in this report we present isolation and characterization (at both molecular and functional levels) of newly discovered bacteriophages specific to several strains of *S. enterica.*

## 2. Results

### 2.1. Isolation of Bacteriophages

Newly discovered bacteriophages were isolated from homogenates of chicken feces, using *S. enterica* serovars Typhimurium 13 and Enteritidis 1392 as hosts, according to a method described in Section 4.3. We isolated 25 different phages which formed five different types of plaques: (i) Turbid with diameter < 1 mm, (ii) turbid with diameter > 1 mm, (iii) clear with diameter < 1 mm, (iv) clear with diameter > 1 mm, and (v) clear with diameter > 1 mm and forming a halo. Two phages were selected for further analysis—the first one isolated using *S.* Typhimurium 13 as a host (bacteriophage named vB_Sen-TO17) and the second one isolated using *S.* Enteritidis 1392 as a host (bacteriophage named vB_Sen-E22).

### 2.2. Plaque and Virion Morphologies

**Figure 1:**
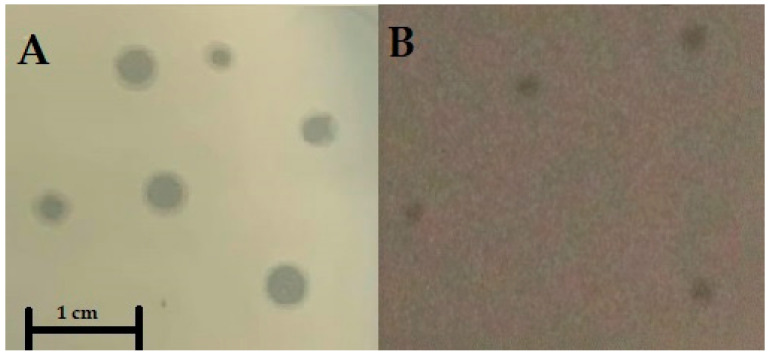
Morphology of plaques of vB_Sen-TO17 (**A**) and vB_Sen-E22 (**B**), formed on a lawn of the *S.* Typhimurium 13 host. A size bar (representing 1 cm) is shown at the bottom of panel A.

The first steps of characterization of newly isolated bacteriophages consisted in determination of morphologies of plaques formed by them and morphologies of virions. Both tested phages formed clear plaques on lawns of the *S*. Typhimurium 13 host; however, vB_Sen-TO17 produced significantly larger plaques (with additional halo) than vB_Sen-E22 (Figure 1 and Table 1).

**Figure 2:**
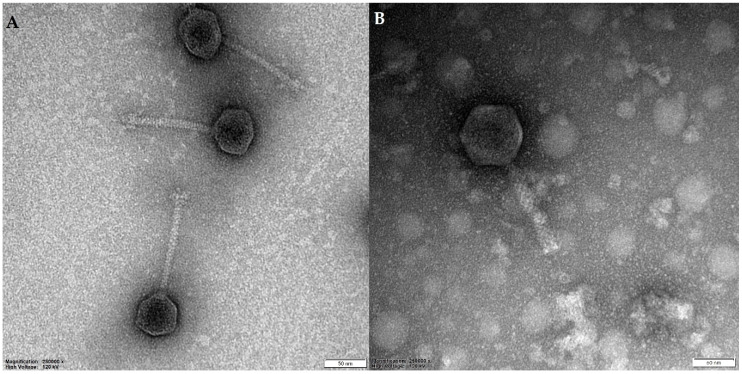
Electron micrographs of virions of vB_Sen-TO17 (**A**) and vB_Sen-E22 (**B**). Size bars (representing 50 nm) are shown at the bottom of each micrograph.

Virion morphology was investigated using electron microscopic methods. Both vB_Sen-TO17 and vB_Sen-E22 are caudate bacteriophages. They have been classified to families *Siphoviridae and Demerecviridae*, respectively, on the basis of morphology of virions and molecular phylogenetic analyses (see Section 2.7). Electron micrographs of virions of both investigated phages are demonstrated in Figure 2, and quantified details of head and tail structures of virions of vB_Sen-TO17 and vB_Sen-E22 are presented in Table 1.

### 2.3. Host Range and Lysogenization Ability

Host ranges of vB_Sen-TO17 and vB_Sen-E22 were determined using various strains of *S. enterica*, representing different serovars. In control experiments, bacterial species either closely related to *S. enterica*, like *Escherichia coli*, or more distant were used. As expected, these experiments confirmed that vB_Sen-TO17 and vB_Sen-E22 are specific for *S. enterica*, while revealing different specificities to various strains, with vB_Sen-E22 effectively infecting significantly more strains than vB_Sen-TO17 (Table 2). Nevertheless, *S. enterica* Gallinarum 74 was infected by vB_Sen-TO17 but not by vB_Sen-E22. These results suggest that both investigated phages might be potentially useful in phage therapy.

To test the ability of vB_Sen-TO17 and vB_Sen-E22 to lysogenize host cells, we performed efficiency of lysogenization assessment as described in Section 4.7. However, none of the tested phages were able to form prophages in 10 tested host strains, *S*. Agona 1408, *S*. Dublin 65, *S.* Enteritidis 64, *S.* Enteritidis 1392, *S*. Heidelberg 16, *S*. Infantis 165, *S*. Newport 50, *S.* Typhimurium 12, *S.* Typhimurium 13, and *S*. Virchow 41 (data not shown as no positive results of the test were observed).

### 2.4. Sensitivity of Bacteriophages to Various Conditions

To test if vB_Sen-TO17 and vB_Sen-E22 are sensitive to different environmental conditions, we tested the survival of virions under various pH (focusing mostly on low pH conditions, which resemble those occurring in a stomach), the presence of various solvents, and the various temperatures of storage and lytic development.

As indicated in Table 3, while vB_Sen-TO17 and vB_Sen-E22 were completely inactivated at pH 2 or lower, significant fractions of viruses could survive in low pH conditions of 2.5 (about 4–8%) or 3.0 (about 55–67%). Moreover, these viruses could also survive in high pH conditions (about 72–85% at pH 10, and about 31–38% at pH 12). These phages were also relatively resistant to ethanol, chloroform, and DMSO, but not to acetone (Table 4). Large fractions (over 55% of virions) of vB_Sen-TO17 and vB_Sen-E22 could survive at temperatures between −80 °C and 60 °C, but not at 95 °C (Table 5). Optimal temperatures for lytic development of both phages were between 37 °C and 42 °C, while vB_Sen-E22 produced its progeny efficiently also at as low a temperature as 20 °C (Table 6). All these results indicate that vB_Sen-TO17 and vB_Sen-E22 are relatively resistant to various environmental conditions, suggesting their potential practical usefulness.

### 2.5. Adsorption Efficiency and Kinatics of Lytic Development

**Figure 3:**
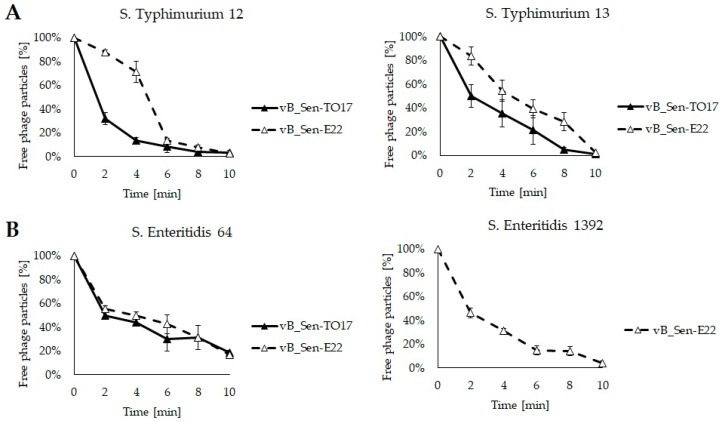
Adsorption rates of phages vB_Sen-TO17 (closed triangles) and vB_Sen-E22 (open triangles) on *S.* Typhimurium (**A**) and *S.* Enteritidis (**B**) at 42 °C. Number of free phage particles at time 0 was used as reference value (100%). Mean values from three independent experiments are shown, with error bars representing SD. Note that *S.* Enteritidis 1392 is resistant to vB_Sen-TO17 (Table 2); thus, experiments with this strain were performed only for vB_Sen-E22.

We found that phages vB_Sen-TO17 and vB_Sen-E22 adsorbed efficiently on cells of various *S. enterica* strains, with above 95% efficiency within 10 min; the exception was the *S.* Enteritidis 64 host on which the adsorption efficiency was about 80% within 10 min (Figure 3). Therefore, this strain appears to be somewhat resistant to its recognition by virions during the first stages of development of phages vB_Sen-TO17 and vB_Sen-E22.

**Figure 4:**
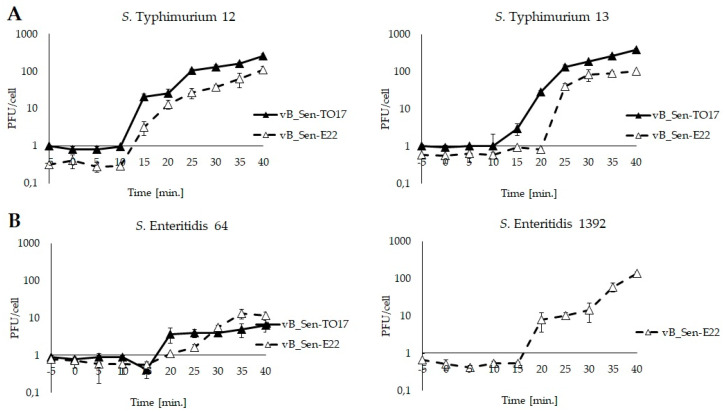
One-step growth experiments with phages vB_Sen-TO17(closed triangles) and vB_Sen-E22 (open triangles) on *S.* Typhimurium (**A**) and *S.* Enteritidis (**B**) at 42 °C; m.o.i. = 0.01. Mean values from three independent experiments are shown, with error bars representing SD. Note that *S.* Enteritidis 1392 is resistant to vB_Sen-TO17 (Table 2); thus, experiments with this strain were performed only for vB_Sen-E22.

Kinetics of phage development have been investigated in one-step growth experiments. As both phages were isolated from chicken feces and were propagated on *S. enterica*, these experiments were performed at 42 °C to resemble conditions occurring in chicken intestine. While efficiency of lytic development was different in various host strains, the eclipse period was between 10 and 20 min, and the development was complete within 30–40 min (Figure 4). Burst sizes of Sen-TO17 and vB_Sen-E22 varied between different host strains, ranging from 9 to 79 progeny phages per cell for vB_Sen-TO17 and from 18 to 64 for vB_Sen-E22 (Table 7). These results indicate that lytic development of both vB_Sen-TO17 and vB_Sen-E22 is effective in various *S. enterica* host strains at 42 °C.

### 2.6. Analysis of Phage Genomes

DNA was isolated from purified virions of vB_Sen-TO17 and vB_Sen-E22 and subjected to sequencing, as described in Section 4.14. Annotations of vB_Sen-TO17 and vB_Sen-E22 genomes are presented in Tables S1 and S2, respectively.

**Figure 5:**
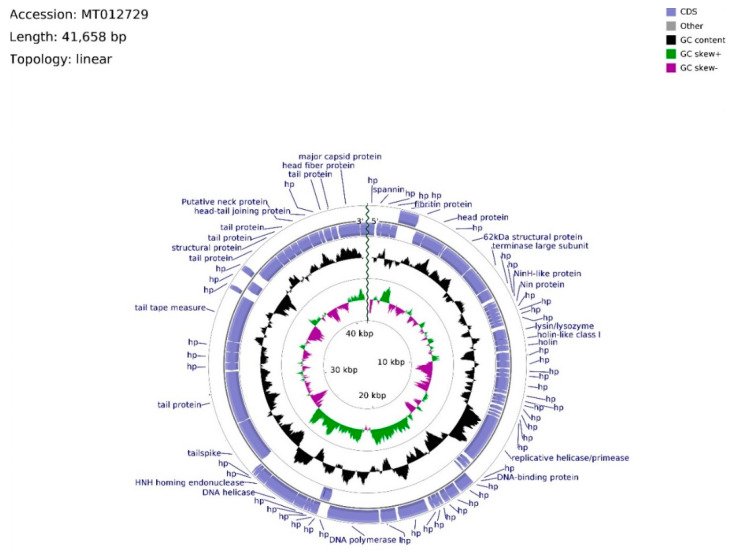
A schematic linear map of phage vB_Sen-TO17 genome (ends of the genome are indicated by the wavy line). The inner rings show genome location, GC skew + (green) and − (purple) and GC content (black). Two the most external rings show identified open reading frames (blue arrows) and results of genome annotation process.

The genome of phage vB_Sen-TO17 (whole sequence deposited in GenBank; accession no. MT012729) consists of 41,658 bps, arranged in a linear topology with an overall GC content of 50.78% (Figure 5). We identified open reading frames (ORFs) putatively coding for 75 proteins, of which 27 were reported previously. The remaining 48 ORFs are described as hypothetical.

Twenty ORFs are spread out on the leading strand, whereas the majority (55) of ORFs are located on the complementary strand. ATG codon predominates among start codons (70 cases), whereas GTG and TTG occur 3 and 2 times, respectively. ORFs initiating with GTG and TTG codons, with the exception of vB_SenTO17_45 (696 bp), have sequence spans ≤ 144 bp, where the average length of sequence in the vB_Sen-TO17 genome is 535 bp. ATG codon is utilized in every ORF with an assigned function. The frequency of observed stop codons is set out as follows: TAA—40, TGA—28, TAG—7. ORFs were divided into four functional groups due to the assigned functions of their putative products: Morphogenesis (15), DNA replication (4), lysis (3), and DNA packing (2). The total number of ORFs in functional groups was excessive due to the domain determination of hypothetical proteins. In consequence, putatively protein-encoding vB_SenTO17_52, bearing the HNH endonuclease domain sequence, was classified within the DNA replication functional group, joined with vB_SenTO17_34, vB_SenTO17_36, vB_SenTO17_46, and vB_SenTO17_53, encoding helicase/primase, DNA-binding protein, DNA polymerase I, and DNA helicase (which start codon overlaps vB_SenTO17_52 stop codon), respectively. DNA replication genes are spread within the 12,966–25,568 bps span. The majority of ORF coding proteins putatively engaged in morphogenesis are located downstream, within the 26,354–41,287 bps region, with the exception of genes encoding fibritin (vB_SenTO17_06), putative head protein (vB_SenTO17_07), and 62 kDa structural protein (vB_SenTO17_09), located at the 2282–5401 bps region. Numerous possible transcription promoters were registered on the complementary strand in the morphogenesis-related region, in relation to the whole genome. The morphogenesis group consists mostly of genes coding for proteins involved in tail assembly (vB_SenTO17_07, vB_SenTO17_57, vB_SenTO17_58, vB_SenTO17_62, vB_SenTO17_66, vB_SenTO17_68, vB_SenTO17_69, vB_SenTO17_73), head (vB_SenTO17_74, vB_SenTO17_75), head–tail joining proteins (vB_SenTO17_70, vB_SenTO17_71), and structural proteins (vB_SenTO17_09, vB_SenTO17_67). Coding DNA Sequences (CDSs) corresponding to lysis proteins are located upstream of the DNA replication span, and occupy positions at 8694–9739 bps. They include ORFs for lysin/lysozyme (vB_SenTO17_19), putative holin-like class I (vB_SenTO17_20), and putative holin (vB_SenTO17_21). Nevertheless, based on nucleotide sequence analysis using the PHACTS algorithm, phage lifestyle was non-confidently classified as temperate. DNA packing CDSs are located within the 5418–6641 bps region, and the 25,565–26,062 bps span consists of genes putatively encoding the terminase large subunit (vB_SenTO17_10) and the HNH homing endonuclease (vB_SenTO17_54). Li’s method analysis suggested that phage vB_Sen-TO17 genome is packaged according to the PAC system. Following ORFs: vB_SenTO17_54, vB_SenTO17_53, and vB_SenTO17_46 are the only sequences located on the leading strand which may code for proteins with the reported function.

**Figure 6:**
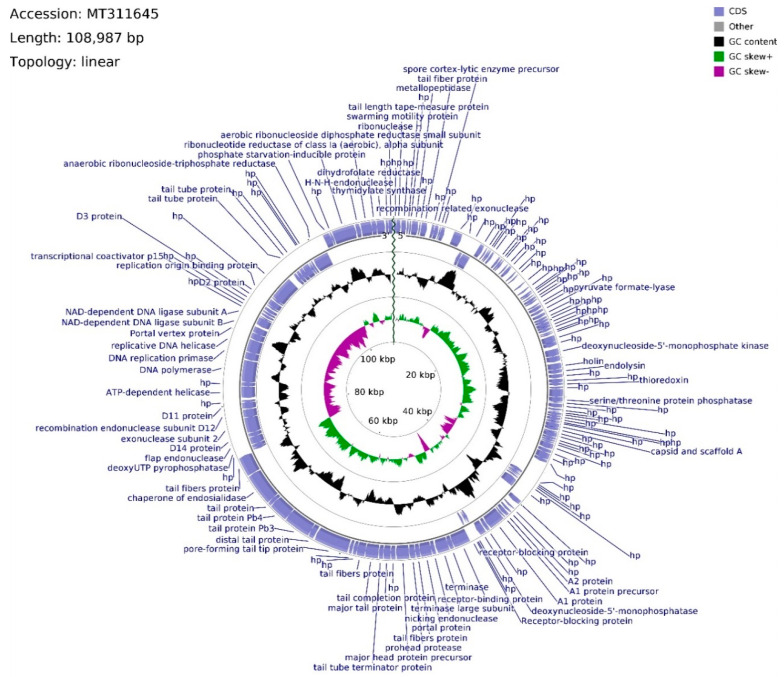
Schematic linear map of phage vB_Sen-E22 genome (ends of the genome are indicated by the wavy line). The inner rings show genome location, GC skew + (green) and − (purple) and GC content (black). The two most external rings show identified open reading frames (blue arrows) and results of genome annotation process.

The genome of phage vB_Sen-E22 (whole sequence deposited in GenBank; accession no. MT311645) consists of 108,987 bp, with overall GC content of 39.21% and linear topology (Figure 6). Determination of ORFs distinguished 158 putative protein-coding genes where 114 were located on the leading strand and 44 were located on the complementary strand. The frequency of start codons is set out as follows: ATG—147, GTG—8, TTG—3. Among termination codons, nucleotide triplet frequencies were set as follows: TAA—124, TGA—25, TAG—8. The functions of 67 ORFs were assigned, whereas 91 remain hypothetical. ORFs with assigned functions were divided into four functional groups: Morphogenesis (22), DNA packing (10), DNA replication (7), and lysis (2). Morphogenesis CDSs are concentrated inside the region of 46,010–74,545 bps, with nine sequences interspersed throughout the vB_Sen-E22 genome, mainly coding for tail-related proteins with the exception of head assembly proteins vB_SenE22_68 and vB_SenE22_131. CDSs engaged in tail protein assembly dominate this genome region (11 CDSs), whereas two head-related CDSs, coding for portal protein (vB_SenE22_100) and major head protein precursor (vB_SenE22_103), are also present there. Phage head putative genes are located within the 50,510–54,248 bps span which is intersected with the tail fibers protein putative gene (vB_SenE22_101) and the sequence putatively encoding prohead protease (vB_SenE22_102). DNA packing genes are spread across the vB_Sen-E22 genome, and they include genes encoding putative nucleases: Endonucleases (vB_SenE22_99, vB_SenE22_120, vB_SenE22_123, vB_SenE22_153), exonucleases (vB_SenE22_12, vB_SenE22_112), and ribonuclease H (vB_SenE22_158).

Apart from ORF for potential nicking endonuclease (vB_SenE22_99), two terminase subunit ORFs are situated on the leading strand which are preceded by CDSs of three receptor-blocking proteins. Using Li’s method, the DNA packaging of this phage can be suggested as operating by the COS mode. ORF for recombination-related exonuclease (vB_SenE22_12) is located upstream of the one for the hypothetical protein bearing PHB domain putatively engaged in phage decision between lytic and lysogenic growth. Within this domain, a Rho-independent terminator is located between vB_SenE22_12 and vB_SenE22_13 CDSs. The gap of the non-coding region encompasses 943 bps, whereas the average length of a gap between coding DNA sequences across the genome is equal to 93 bps. ORFs coding for proteins putatively involved in the process of DNA replication, located on the complementary strand, are assembled in a tile-like manner at the 80,720–90,335 bps region, with the putative replication origin binding protein ORF (vB_SenE22_141) situated upstream of the CDS conglomerate. vB_SenE22_141 (93,024–95,813 bp) overlaps with the vB_SenE22_140 hypothetical protein gene, bearing two transcription terminators starting at positions 117 bp and 183 bp inside the 234 bps long CDS. A transcription terminator can also be found within vB_SenE22_141 CDS, and downstream from the DNA ligase subunit B gene (vB_SenE22_132), which overlaps the A subunit ORF (vB_SenE22_133). The DNA replication tile is interlaced with the ORF encoding uncharacterized protein and the Portal vertex (vB_SenE22_131), belonging to the morphogenesis functional group. Between those sequences, there are ORFs for DNA helicase, DNA replication primase, and DNA polymerase, which are probably transcribed as two operons, as suggested by an overlap between start and stop codons with a 62 bps gap between vB_SenE22_129 and vB_SenE22_128. Based on the sequence analysis with PHACTS, this phage was non-confidently classified as lytic. Putative holin and endolysin CDSs (vB_SenE22_50 and vB_SenE22_51, respectively), representing genes coding for proteins involved in host cell lysis, overlap at positions 24,499–25,565 bps, shifting the probability of the lifestyle classification.

### 2.7. Phylogenetic Analyses

**Figure 7:**
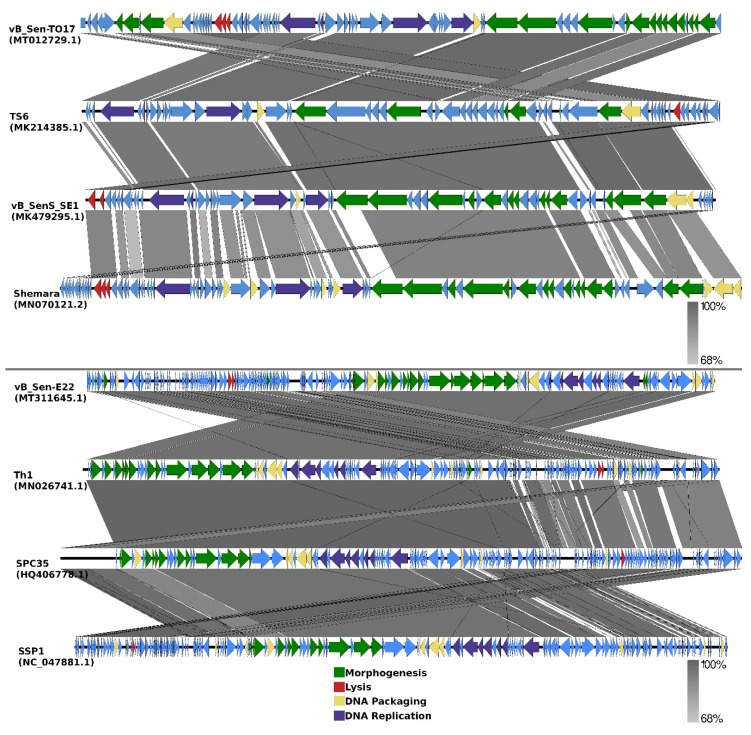
EasyFig output image of the genomic comparison between phages vB_Sen-TO17 and vB_Sen-E22, and the most closely related phages. Phage genomes are presented by linear visualization with coding regions shown as arrows. Selected open reading frames are colored in relation to their functions. The percentage of sequence similarity is indicated by the intensity of the gray color.

Comparisons of organizations of genomes of phages vB_Sen-TO17 and vB_Sen-E22 to genomes of the most related bacteriophages (according to DNA sequence similarities of whole genomes) are indicated as EasyFig in Figure 7.

**Figure 8:**
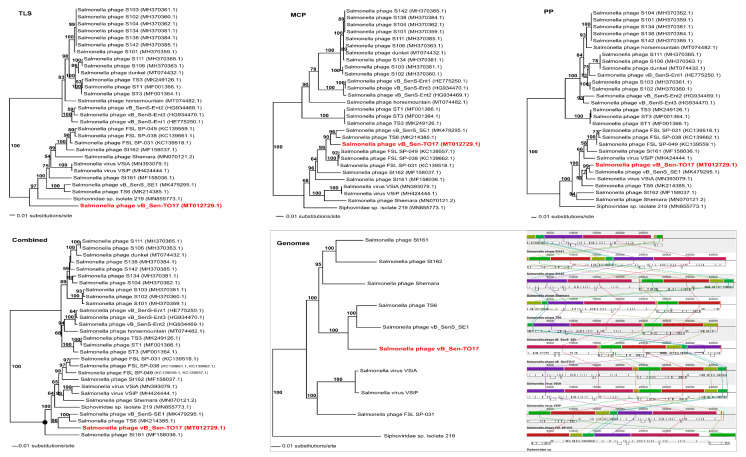
Neighbour-joining phylogenetic trees showing the phylogenetic position of phage vB_Sen-TO17 (in red color) within Cornellvirus based on the sequences coding for terminase large subunit (TLS), major capsid protein (MCP), portal protein (PP), their combined nucleotide sequences (Combined), as well as on the whole-genome analysis (Genomes). The reference sequences were collected from the NCBI database. The tree was constructed using PAUP *. Bootstrap value > 50%, calculated based on 1000 resamplings, is shown above the branches.

To analyse phylogenetic relationships between phage vB_Sen-TO17 and other viruses, we have compared the nucleotide sequences of the gene of the terminase large subunit and the nucleotide sequences of two additional markers such as genes encoding the portal protein and the major capsid protein of vB_Sen-TO17 with the respective sequences of other phages (Figure 8). The use of only the large terminase subunit gene sequence was insufficient to show the actual phylogenetic position of vB_Sen-TO17. The analysis of the nucleotide sequence of the gene coding for portal protein indicated that phage vB_Sen-TO17 is a sister to phage vB_SenS_SE1 (MK479295.1) with high bootstrap support (BS = 99). On the other hand, as shown in Figure 8, the analysis of the major capsid protein gene together with the combined analysis of three marker genes’ sequences (*TLS, PP, MCP*) indicated a close relationship of vB_Sen-TO17 to phages vB_SenS_SE1 (MK479295.1) and TS6 (MK214385.1) with high bootstrap support of BS = 90 and BS = 96, respectively. Both phages belong to the family *Siphoviridae*, genus *Cornellvirus*. Sequence similarity searches of these phages demonstrated very high level of genome sequence identity with vB_Sen-TO17 (~96% and ~98%, respectively). The above results were confirmed by the whole-genome phylogenetic analysis and the whole-genome alignments constructed using the Mauve algorithm (see Section 4.15 for details), which indicated a high level of homology between these genomes (Figure 8). Nevertheless, one should note some differences in the trees topology obtained with the use of various methods presented above, for example in the position of Shemara phage (MN070121.2).

**Figure 9:**
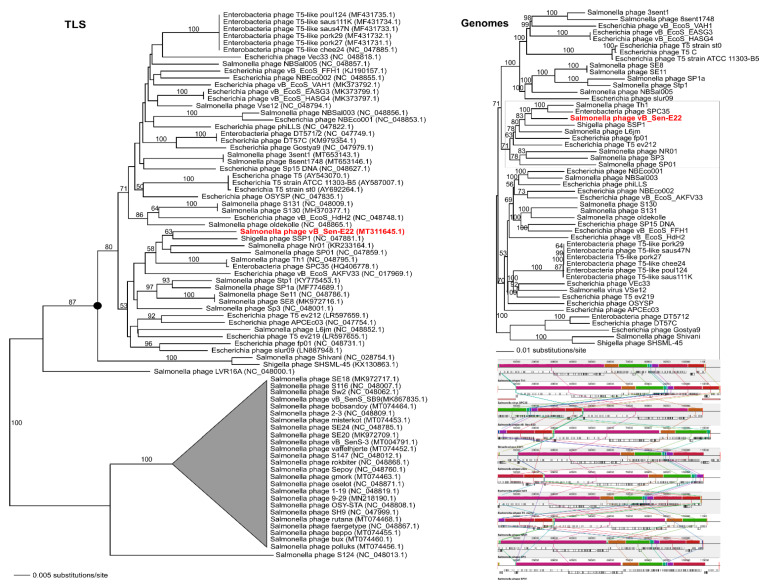
Neighbour-joining phylogenetic tree showing the phylogenetic position of phage vB_Sen-E22 (in red color) within Tequintavirus, based on the sequence coding for terminase large subunit (TLS), as well as on the whole-genome analysis (Genomes). The reference sequences were collected from the NCBI database. The tree was constructed using PAUP *. Bootstrap value > 50%, calculated based on 1000 resamplings, is shown above branches.

To analyse phylogenetic relationships between phage vB_Sen-E22 and other viruses, we compared the nucleotide sequence of the gene coding for the large terminase subunit of vB_Sen-E22 with the sequences of genes of the large terminase subunit of other phages. As shown in Figure 9, the sequence of the large terminase subunit gene of vB_Sen-E22 indicates its relationship to *Shigella* phage SSP1 (NC_047881.1) with low bootstrap support BS = 63. On the other hand, the sequence similarity searches revealed that these phages show a very high level of genome sequence identity (~97%). The whole genome sequence analysis indicated that the phage vB_Sen-E22 is a sister to the phages Th1 (NC_048795.1) and SPC35 (HQ406778.1), with the highest BS = 100 in both cases (Figure 9). Phages SSP1, Th1, and SPC35 belong to the family *Demerecviridae*, genus *Tequintavirus*. Sequence similarity searches between Th1, SPC35, and phage vB_Sen-E22 demonstrated a very high level of identity (~97% when comparing vB_Sen-E22 with Th1, and ~96% when comparing vB_Sen-E22 with SPC35). The whole-genome alignments constructed using the Mauve algorithm (see Section 4.15 for details) also revealed a high level of homology between these genomes (Figure 9). Therefore, for vB_Sen-E22, the single marker gene phylogenetic analysis was insufficient and did not reflect the actual genetic position of this phage, probably due to mosaicism of phage genomes and a horizontal gene transfer. Nevertheless, the whole genome sequence phylogenetic analysis allowed us to obtain reliable results, leading to the proposal that vB_Sen-E22 belongs to the family *Demerecviridae*, genus *Tequintavirus*.

## 3. Discussion

Among foodborne pathogens, *S. enterica* is one of the most frequently occurring infectious agents, and a majority of infections arise from contamination of poultry-derived products [1,2]. Antibiotic resistance occurs commonly in this bacterium; thus, alternative methods of eradication of this pathogenic microorganism are required [3]. One might consider that anti-*Salmonella* phage therapy could be a potential option to eliminate *S. enterica* from poultry, including both gastrointestinal tract of birds and poultry-derived products. On the other hand, effective phage therapy requires specific conditions to be applied in practice [35]. Since each phage is specific to particular serovars or strains, a large collection of bacteriophages is required for effective phage therapy. Since temperate phages can lysogenize bacterial cells, virulent bacteriophages are preferred to eradicate pathogenic bacteria. It is also crucial that genomes of bacteriophages used in phage therapy are devoid of any genes coding for toxins or other proteins which are detrimental for humans or animals. Finally, phages revealing rapid and effective development are preferred in phage therapy as they may eliminate target bacteria in a relatively short time. Therefore, isolation and characterization of a large set of different bacteriophages infecting *S. enterica* is desirable if phage therapy against this bacterium can be used in practice.

In this report, we describe isolation and characterization of two bacteriophages infecting *S. enterica*. The viruses (named vB_Sen-TO17 and vB_Sen-E22) were newly isolated from chicken feces and both molecular and functional characterization has been performed. Despite considerable differences in their genomes, including various sizes (41,658 bp for vB_Sen-TO17 and 108,987 bp for vB_Sen-E22) and organizations (Figure 7), both phages do not encode any homologs of known toxins or toxin motifs, reveal effective adsorption on host cells, and develop effectively in *S. enterica* cells giving burst sizes between 9 and 79 PFU/cell, depending on the host strain, at 42 °C (a temperature resembling that occurring naturally in chicken body). Moreover, their virions are relative stable under various conditions, including low pH (Table 3), presence of various solvents (Table 4) and different temperatures (Table 5). Although host ranges of tested phages are different, when considered together, their spectrum of sensitive *S. enterica* serovars is relatively large (Table 2). These features predispose vB_Sen-TO17 and vB_Sen-E22 to be used in phage therapy procedures.

Despite vB_Sen-E22 genome is about 2.6 times larger than that of vB_Sen-TO17 (108,987 bps vs. 41,658 bps), a diameter of the head of the former phage is only 10 nm bigger than that of the latter virus (58 vs. 48 nm). Nevertheless, when calculating volumes of heads of these bacteriophages, one can estimate that they are about 101.8 thousand nm^3^ and about 57.7 thousand nm^3^ for vB_Sen-E22 and vB_Sen-TO17, respectively. Therefore, the genome of vB_Sen-E22 must be only moderately more densely packaged inside the head than that of vB_Sen-TO17.

Genomic analyses, performed with using the PHACTS algorithm (see Section 4.14), suggested that vB_Sen-TO17 might be a temperate phage. On the other hand, absence of genes with clear homology to those coding for integration and immunity proteins makes such a possibility doubtful. Moreover, the prediction that this might be a temperate phage was not confirmed by experimental results. The vB_Sen-TO17 virus forms clear plaques (Figure 1 and Table 1) and was found to be unable to lysogenize host cells under laboratory conditions. Therefore, we assume that a halo visible around plaques of this phage may arise from activities of lytic enzymes which if released from lysed cells and remining relatively stable in the agar, might cause inhibition of growth of uninfected bacteria neighboring lysed ones. Since vB_Sen-TO17 encodes 3 lytic enzymes, lysin/lysozyme (vB_SenTO17_19), putative holin-like class I (vB_SenTO17_20), and putative holin (vB_SenTO17_21), such hypothesis appears to be of high probability. Therefore, we conclude that it is a virulent rather than temperate phage, while genomic analyses might indicate features of its ancestors, and corresponding genes may be either inactive now or play different roles in phage development.

Bacteriophage vB_Sen-E22 also forms clear plaques but without halo (Figure 1 and Table 1). Moreover, its genome is characteristic for virulent phages, confirming that this virus is unable to form prophages in infected bacteria.

Molecular phylogenetic studies were somewhat complicated in the case of vB_Sen-TO17, as the analysis of the gene encoding large terminase subunit (classical molecular marker for such analyses in studies on bacteriophages) was not sufficient to determine the actual phylogenetic position of this phage. Nevertheless, comparisons of sequences of two additional genes allowed us to indicate that vB_Sen-TO17 is closely related to two other phages, vB_SenS_SE1 and TS6, which belong to the family *Siphoviridae* and genus *Cornellvirus*. Since sequence similarity searches indicated a high level of identity to vB_Sen-TO17, about 96% and about 98%, respectively, we propose to classify the newly isolated phage to family *Siphoviridae* and genus *Cornellvirus*. This conclusion was corroborated by whole-genome phylogenetic analysis.

Phylogenetic analyses of vB_Sen-E22 were also not obvious and easy, as similarity of its large terminase subunit gene did not reveal high identity with any other phage. The analyses indicated its relationship to *Shigella* phage SSP1; however, the bootstrap support was relatively low (BS = 62). Nevertheless, since whole genome sequence identity between these two phages is at the level of 97%, and such a level of similarity occurs also when comparing two other phages closely related to vB_Sen-E22, Th1, and SPC35, which belong to family *Demerecviridae* and genus *Tequintavirus*, we propose to classify vB_Sen-E22 to the same family and genus.

In conclusion, our studies, presented in this report, indicate that newly isolated and characterized bacteriophages vB_Sen-TO17 and vB_Sen-E22 can be used for further studies on anti-*Salmonella* phage therapy, particularly for eradication of *E. enterica* from poultry.

## 4. Materials and Methods

### 4.1. Bacterial Strains

Strains of *S. enterica*, used in this study, come from the National Salmonella Center at Medical University of Gdansk (Gdansk, Poland). Strains of *Proteus vulgaris, Citrobacter freundii, Enterococcus faecalis,* and *Escherichia coli* come from the Department of Molecular Biology of the University of Gdansk collection of microorganisms (Gdansk, Poland). *Staphylococcus aureus* strain 6538™, *Lactococcus lactis* 49032^TM^ and *Lactobacillus acidophilus* 314^TM^ come from ATCC.

### 4.2. Bacterial Culture Conditions

Bacteria were cultured at 37 °C or 42 °C. LB-medium (BioShop, Burlington, ON, Canada) was used to cultivate Gram-negative bacteria. For *S. aureus*, *E. faecalis, L. lactis,* and *L. acidophilus,* a BHI medium (Graso Biotech, Starogard Gdański, Poland) was used. Bacteriological agar (BioShop, Burlington, ON, Canada) at a final concentration of 1% was used in solidified media (LB-agar or BHI-agar). *L. lactis* and *L. acidophilus* were cultivated under microaerophilic conditions using the GenBox microaer system (BioMérieux, Marcy l’Etoile, France).

### 4.3. Isolation and Purification of Phages vB_Sen-TO17 and vB_Sen-E22

Chicken feces were suspended in an LB medium at a 1:10 ratio, homogenized using hand homogenizer (GenoPlast Biochemicals, Rokocin, Poland), and incubated overnight at 37 °C (Heraeus B-12, Kendro Laboratory Products, Langenselbold, Germany). The samples were then centrifuged at 6000× g for 20 min at 4 °C (Avanti JXN-26, rotor JS-13.1, Beckman Coulter, Indianapolis, USA), and supernatants were collected and filtered through a 0.22 µm syringe filter (Millex-GP, Sigma-Aldrich, USA). Ten-fold dilutions were prepared in an LB medium and 100 µL of each dilution was mixed with 200 µl of bacterial host culture and 4 mL of LB with 0.7% agar. The mixture was poured onto plates containing 20 mL of LB-agar. The double-layer agar plates were incubated at 42 °C (BF 53, BINDER GmbH, Tuttlingen, Germany) overnight and then scanned for plaques. Different looking plaques were then collected, transferred to 10 mL of freshly diluted (1:100 ratio) host strain culture, and incubated with shaking at 155 rpm for 3 h at 42 °C (OLS 200, Grant Instruments, Sherpeth, UK). Obtained lysates were treated with 5 mL chloroform (Alchem, Torun, Poland), centrifuged (4000× g, 10 min, 4 °C), and filtered through 0.22 µm filter. The lysates were then titrated on double-layer agar plates. The plates were incubated overnight at 42 °C and then scanned for uniform plaques. The purity of lysates was also checked using electron microscopy.

### 4.4. Phage Propagation

A 10 mL amount of bacterial host culture, grown overnight in LB medium, was added to 1 L of LB and incubated at 37 °C with agitation at 150 rpm. At OD_600_ = 0.15 (measured by using SmartSpec PLUS, BIO-RAD, CA, USA) bacteria were infected with phages at a multiplicity of infection (m.o.i.) of 0.5 and incubated at 37 °C until lysis occurred. For phage purification, polyethylene glycol (PEG) 8000 (BioShop, Burlington, Ontario, Canada) was added to the final concentration of 10% and stirred (Carl Roth, Karlsruhe, Germany) overnight at 4 °C. The precipitate was collected by centrifugation at 10,000× g for 30 min at 4 °C (Avanti JXN-26, rotor JLA-8000, Beckman Coulter, IN, USA) and suspended in 0.89% NaCl (Alchem, Torun, Poland). PEG 8000 was removed by adding 2 mL of chloroform and centrifugation at 4000× g for 15 min at 4 °C (Avanti JXN-26, rotor JS-13.1, Beckman Coulter, IN, USA). The procedure was repeated until no PEG 8000 precipitate could be observed.

### 4.5. Electron Microscopy

Phages were purified by centrifugation using CsCl (Sigma Aldrich, MO, USA) density gradient as described previously [36]. Transmission electron microscopy analysis of phage capsids was performed in the Laboratory of Electron Microscopy, Faculty of Biology, University of Gdansk, Gdansk, Poland. Virions were negatively stained with uranyl acetate (VWR International Ltd., Radnor, PA, USA) and then micrographs were taken under a Tecnai G2 Spirit BioTWIN electron microscope (FEI, Thermofisher Scientific, OR, USA).

### 4.6. Plaque Morphology Assessment

The plaque morphology analysis of bacteriophages was performed using *S.* Typhimurium 13 as a host. Ten-fold dilutions of phage lysate were prepared in 0.89% NaCl; 200 µL of overnight host culture were mixed with 10 µL of an appropriate dilution of phage lysate and added to 4 mL of LB with 0.7% agar. The mixture was poured onto plates containing 20 mL of LB agar. The double-layer agar plates were incubated at 42 °C for 16 h. Plaque morphology and diameter were determined.

### 4.7. Determination of Phage Host Range and Efficiency of Lysogenization

Host range of bacteriophages was determined using the spot-test method described previously [34], with some modifications. Ten-fold dilutions of phage stocks were prepared in 0.89% NaCl; 10 µL of the appropriate dilution was mixed with 200 µL of overnight bacterial culture and 4 mL of LB with 0.7% agar. Plates were incubated overnight at 37 °C and then scanned for plaques.

For lysogenization experiments, *S*. Agona 1408, *S*. Dublin 65, *S.* Enteritidis 64, *S.* Enteritidis 1392, *S*. Heidelberg 16, *S*. Infantis 165, *S*. Newport 50, *S.* Typhimurium 12, *S.* Typhimurium 13, and *S*. Virchow 41 were cultivated to OD_600_ = 0.2 at 42 °C. Then, a sample of 1 mL of bacterial culture was centrifuged (2000× *g*, 10 min, 4 °C) and the pellet was resuspended in 1 mL of 0.5 × LB medium. Following incubation for 5 min at 30 °C, phage lysate was added to m.o.i. = 1. In the control variant of the experiment, 0.5 × LB was added instead of phage lysate. Bacteria were incubated for 3 h at 30 °C, and then centrifuged (4000× *g*, 5 min, 4 °C) in order to remove free phage particles. Supernatant was discarded, and the pellet was resuspended in a fresh LB medium. Serial dilutions were prepared in 0.89% NaCl and 30 µL of each dilution was spread onto LB plates. After 24 h incubation at 30 °C, 96 colonies were passaged separately, each in a well of a 96-well plate with 150 µL of LB medium. The plates were incubated with shaking at 42 °C until bacteria culture reached OD_600_ = 0.2.

For estimation of efficiency of lysogenization, mitomycin C was added (to a final concentration of 1 µg/mL) to 150 µL of bacterial culture derived from a single tested colony (this antibiotic had been demonstrated previously to induce prophages in *S. enterica* [37,38,39]). The plates were then incubated for 3 h. Afterwards, 10 µL of chloroform was added, the plates were centrifuged (2000× *g*, 10 min, 4°C), and 5 µL of water phase was spotted onto double-layer LB agar plates. The plates were incubated overnight at 42 °C and then scanned for plaques. A colony was determined as lysogenic if plaques were formed on bacterial lawn. The efficiency of lysogenization was determined as a percent of lysogens among all tested bacterial colonies. The experiment was performed in triplicate.

In order to test the resistance to superinfection, 50 µL of bacterial culture was mixed with 4 mL of 0.7% LB agar and poured onto an LB agar plate. Then, 2.5 µL of phage lysate was spotted on top of it. The plates were incubated overnight at 42 °C and scanned for plaques. If plaques were not visible, the clone was recognized as resistant to phage. The resistance to infection was determined as a percent of bacteria not infected by the phage. The experiments were performed in triplicates.

### 4.8. Phage Sensitivity to Various Conditions

Sensitivity of phages to low pH conditions was performed as described previously [21]. Briefly, 100 µL of phage lysate were added to 900 µL of LB at pH 1.8, 2.0, 2.2, 2.5, 2.8, 3.0, 10.0, and 12.0, with pH 7.0 used as a control variant. The pH was adjusted by addition 1 M HCl (Alchem, Torun, Poland) or 1 M NaOH (Alchem, Torun, Poland) and measured using a pH meter (pH 50+ DHS, Giorgio Bormac, Carpi, Italy). Samples were mixed and incubated for 1 h at 42 °C. Afterwards, samples were serially diluted and overlaid on top of LB agar plates with the top agar containing 200 µL of overnight bacterial culture. Plates were incubated overnight at 37 °C and then scanned for plaques. Susceptibility to disinfectants, liquid media, and buffers were performed in accordance with a procedure described previously [34,37]. Briefly, 100 µL of phage lysate was added to 900 µL of tested solution. The samples were mixed and incubated for 1 h at 25 °C or for 24 h at 37 °C for DMEM (Thermo Fisher Scientific Inc., Paisley, UK), supplemented with 10% fetal bovine serum (Thermo Fisher Scientific Inc., Paisley, UK) and 1% antibiotic/antimycotic solution (Sigma Aldrich Co. LLC., St. Louis, MO, USA). The experiments were performed in triplicates.

### 4.9. Phage Sensitivity to Different Temperatures

Phage survivability at different temperatures was assessed in accordance with previously described protocol [34]. Phages were diluted in 0.89% NaCl to a final concentration of 10^9^ PFU/mL and then incubated at temperature −80 °C (Revco ULT-1790-10V, Thermo Fisher Scientific, OR, USA), −20 °C, 4 °C, 25 °C, 37 °C, 42 °C, 60 °C, or 95 °C for a given period of time. Following the incubation, 10-fold dilutions were prepared in 0.89% NaCl and overlaid on top of double agar plates. The plates were incubated at 42 °C overnight and then scanned for plaques. The percent of surviving phages was calculated as a ratio of surviving phages to phage titer before incubation. The experiments were done in triplicates.

### 4.10. Efficiency of Phage Plating at Different Temperatures

The analysis of phage titration dependence on temperature was performed in accordance with previously described protocol [34]. Briefly, 10-fold dilutions of phage lysates were prepared in 0.89% NaCl and overlaid on top of double agar plates. The plates were incubated at 20 °C, 25 °C, 37 °C, and 42 °C overnight. The efficacy of phage plating was assessed by comparison of phage titers obtained at different temperatures. The experiments were performed in triplicates.

### 4.11. Efficiency of Phage Adsorption

An adsorption assay was performed according to the protocols described previously [40], with some modifications. Overnight cultures of bacterial strains were diluted 1:100 in fresh LB medium and incubated with shaking at 42 °C until OD_600_ = 0.2 was reached. Then, 2 mL samples were centrifuged at 2000× *g*, 5 min, 4 °C (MiniSpin Plus, Eppendorf, Hamburg, Germany) and the pellet was suspended in 1 mL of fresh LB medium. After 10 min incubation at 42 °C, phages were added to final m.o.i. of 0.1. At given time points, 100 µL samples were collected and centrifuged at 6000× *g* for 30 s. Ten-fold dilutions were prepared and overlaid on top of double-layer agar plates containing 0.7% LB agar with 200 µL of bacterial host culture. The number of viruses mixed with bacterial host cells at time 0 was considered 100% non-adsorbed phages. Other values were compared to this sample. The experiment was performed in triplicates.

### 4.12. One-Step Growth Experiments

One-step growth experiments were performed as described previously [21], with some modifications. Bacteria were grown at 42 °C until reaching optical density of OD_600_ = 0.1. Then, 10 mL of bacterial culture were centrifuged (4000× *g*, 10 min, 4 °C) (Avanti JXN-26, rotor JS-13.1, Beckman Coulter, Indianapolis, USA), and the pellet was suspended in 1 mL of LB medium at 4 °C. Phages were added to the host culture at m.o.i. = 0.1 and allowed to adsorb for 5 min at 42 °C. The mixture was centrifuged at 4,000× *g* for 10 min at 4 °C to remove unadsorbed phage particles. After centrifugation, 50 µL of phage-bacteria mixture was added to 20 mL of LB medium (time 0) and cultivated at 42 °C. The number of infective centers was estimated from samples taken 1 min, 2.5 min, and 5 min after infection, by mixing 10 µL of sample with 200 µL of overnight bacterial culture and 4 mL of 0.7% LB agar. Samples (100 µL each) were collected at given temperatures, mixed with 50 µL of chloroform, cleared by centrifugation (6000× *g*, 30 sec) (MiniSpin Plus, Eppendorf, Hamburg, Germany), and titrated to determine the number of PFU/mL. Simultaneously, 100 µL samples were collected, centrifuged immediately (6000× *g*, 30 s), and titrated. The plates were incubated at 42 °C overnight. The experiment was performed in triplicates. Burst size was calculated as the ratio of phage titer from samples untreated with chloroform to the number of infection centers. Total phage yield was calculated as the ratio of phage titer from chloroform-treated samples to the number of infection centers.

### 4.13. Phage DNA Isolation

Phage lysate, purified as described in Section 4.5, was treated with DNase I (1 U/μL; Thermo Fisher Scientific Inc., Paisley, UK) and RNase A (5 µg/µL; Thermo Fisher Scientific Inc., Paisley, UK) to degrade bacterial nucleic acids. To digest the exogenous DNA and RNA, the mixture was incubated for 30 min at 37 °C. Then, DNase I and RNase A were inactivated by heating to 95 °C and 5 min incubation. Genomic DNA of phages was isolated with a MasterPure™ Complete DNA and RNA Purification Kit (Epicentre Biotechnologies, WI, USA) in accordance with the manufacturer’s guidelines.

### 4.14. Genomic Analysis of Phages vB_Sen-E22 and vB_Sen-TO17

Genomes of vB_Sen-E22 and vB_Sen-TO17 phages were sequenced using the Whole Genome Shotgun (WGS) strategy and run on the MiSeq Illumina platform. Samples for next-generation sequencing were prepared according to the NEBNext DNA Library Prep Master Mix Set for the Illumina manual with random selection. The read length interval ranged from 36 to 251 bp. Raw reads were deposited in SRA databases under BioProject ID: PRJNA671789.

Annotation and sequence analysis was conducted as described previously [21]. Briefly, annotation was based on open reading frame prediction obtained via the RASTtk toolkit [41] and the Prokka suite containing Prodigal [42] where points of difference were settled by analysis of Ribosome Binding Sites (RBS) 4-12 nucleotides upstream of start codons. Putative functions of ORFs were assessed employing the Nucleotide Collection (nr/nt) database with the CDD database [43], 97 Salmonella phage genomes from pVOGs database (http://dmk-brain.ecn.uiowa.edu/pVOGs/, last accessed on 16 November 2020), and the HMMer Reference Proteomes database [44]. Phage-specific promoters were predicted by using the Neural Network Promoter Prediction NNPP method (http://www.fruitfly.org/seq_tools/promoter.html, last accessed on 16 November, 2020). To find Rho-independent terminators in nucleic acid sequences, the Arnold tool was employed [45]. Classification of phages lifestyle was predicted using PHACTS [46]. For phage genome termini analysis, packing mode and topology of viral genomes PhageTerm was employed [47]. To create maps of viral genomes, the CGView Comparison Tool [48] was used, with additional GC skew and GC content analysis. Comparison of genomes of related phages was performed using the EasyFig program (http://mjsull.github.io/Easyfig/files.html, last accessed on 14 August 2020).

### 4.15. Phylogenetic Analysis of Phages vB_Sen-E22 and vB_Sen-TO17

To estimate the phylogenetic positions of the newly isolated phages vB_Sen-E22 and vB_Sen-TO17, the nucleotide sequences of the terminase large subunit gene (genetic marker for the order Caudovirales) were compared with the sequences of other reference bacteriophages that were deposited in the NCBI database. We have also compared the nucleotide sequences of genes coding for major capsid protein and portal protein of vB_Sen-TO17. DNA sequences were translated to amino acid, then aligned and adjusted by eye using Seaview [49]. Additionally, we conducted the whole-genome sequence phylogenetic analyses for both phages. Multiple genome alignments were conducted through the progressive Mauve algorithm [50]. Each subalignment generated by Mauve was adjusted by eye and concatenated using Seaview [49]. All matrices were analyzed using PAUP * (Phylogenetic Analysis Using Parsimony * and Other Methods) version 4.0a [51]. The optimality criterion was set to distance using the Neighbour-Joining algorithm (NJ). The p-distance was used based on a previously published recommendation [52]. The robustness of the tree topology was assessed by bootstrap analyses based on 1000 replicates.

## Figures and Tables

**Table 1:** Morphological characteristics of phages vB_Sen-TO17 and vB_Sen-E22. Experiments were performed using *S*. Typhimurium 13 as a host.

Phage Name	Plaque Morphology	Capsid Morphology		
		Head Diameter	Tail Length	Tail Width
vB_Sen-TO17	Clear, Ø 2.3–2.5 mm, with halo	48 ± 5 nm	121 ± 8 nm	10 ± 3 nm
vB_Sen-E22	Clear, Ø 0.8–1.0 mm	58 ± 3 nm	88 ± 6 nm	12 ± 2 nm

**Table 2:** Host range of phages vB_Sen-TO17 and vB_Sen-E22.

Bacterial Strain	Efficiency of Plating (%) *	
	vB_Sen-TO17	vB_Sen-E22
*Citrobacter freundii*	<0.01	<0.01
*Enterococcus faecalis* 230	<0.01	<0.01
*Enterococcus faecalis* 243	<0.01	<0.01
*Escherichia coli* C600	<0.01	<0.01
*Escherichia coli* MG1655	<0.01	<0.01
*Lactobacillus acidophilus* 314^TM^	<0.01	<0.01
*Lactococcus lactis* 49032^TM^	<0.01	<0.01
*Proteus vulgaris*	<0.01	<0.01
*Salmonella enterica* Agona 1408	<0.01	<0.01
*Salmonella enterica* Bovismorbificans 300	<0.01	<0.01
*Salmonella enterica* Choleraesuis 1439	<0.01	<0.01
*Salmonella enterica* Choleraesuis 34	<0.01	68.49 ± 7.38
*Salmonella enterica* Choleraesuis var. Kunzendorf 37	<0.01	31.72 ± 2.55
*Salmonella enterica* Derby 20	<0.01	<0.01
*Salmonella enterica* Dublin 65	<0.01	85.94 ± 6.12
*Salmonella enterica* Enteritidis 1392	<0.01	100.00 ± 3.16
*Salmonella enterica* Enteritidis 64	39.27 ± 4.87	65.66 ± 5.18
*Salmonella enterica* Gallinarum 74	86.72 ± 4.55	<0.01
*Salmonella enterica* Hadar 1748	<0.01	<0.01
*Salmonella enterica* Heidelberg 16	<0.01	91.83 ± 6.82
*Salmonella enterica* Infantis 155	<0.01	<0.01
*Salmonella enterica* Kentucky 1368	<0.01	<0.01
*Salmonella enterica* Newport 50	<0.01	18.25 ± 6.51
*Salmonella enterica* Newport 51	<0.01	<0.01
*Salmonella enterica* Saintpaul 435	<0.01	<0.01
*Salmonella enterica* Senftenberg 87	<0.01	56.68 ± 4.39
*Salmonella enterica* Stanley 15	<0.01	8.48 ± 1.32
*Salmonella enterica* Thompson 39	<0.01	<0.01
*Salmonella enterica* Typhimurium 12	100.00 ± 3.22	97.88 ± 4.13
*Salmonella enterica* Typhimurium 13	100.00 ± 2.18	100.00 ± 6.43
*Salmonella enterica* Virchow 41	<0.01	85.4 2 ± 2.77
*Staphylococcus aureus* 6538™	<0.01	<0.01

* Efficiency of plating was calculated on the basis of three independent experiments, and mean values ± SD are shown. Results obtained with *S.* Typhimurium 13 were considered as 100%, and other values reflect these results.

**Table 3:** Susceptibility of phages vB_Sen-TO17 and vB_Sen-E22 to different pH.

Phage Name	Phage Survivability in Studied Conditions (Relative Phage Titer in %)								
	pH 1.8	pH 2.0	pH 2.2	pH 2.5	pH 2.8	pH 3.0	pH 7.0 *	pH 10.0	pH 12.0
vB_Sen-TO17	<0.01	<0.01	11.11 ± 2.87	7.78 ± 2.21	18.89 ± 3.46	66.67 ± 7.15	100	85.41 ± 5.66	38.75 ± 3.89
**vB_Sen-E22**	<0.01	<0.01	<0.01	3.89 ± 0.95	33.33 ± 6.17	55.56 ± 8.55	100	72.43 ± 9.22	31.55 ± 4.72

Values obtained at pH 7.0 (marked as *) were assessed as 100% and other values reflect these controls.

**Table 4:** Survivability of phages vB_Sen-TO17 and vB_Sen-E22 at different solvents, media, and buffers.

Phage Name	Phage Survivability in Studied Conditions (Relative Phage Titer in %)						
	70% Ethanol	Chloroform	90% Acetone	10% DMSO	DMEM	10% SDS	0.89% NaCl *
**vB_Sen-TO17**	42.63 ± 4.22	83.33 ± 5.37	0.88 ± 0.07	66.42 ± 7.15	100 ± 3.16	34.62 ± 7.16	100
vB_Sen-E22	8.75 ± 1.27	79.81 ± 6.42	0.23 ± 0.04	76.63 ± 6.85	100 ± 4.28	27.81 ± 4.21	100

Values obtained for 0.89% NaCl (marked as *) were assessed as 100% and other values reflect these controls.

**Table 5:** Survivability of phages vB_Sen-TO17 and vB_Sen-E22 at different temperatures.

Phage Name	Phage Survivability at Different Temperatures (Relative Phage Titer in %)							
	−80 °C (24h)	−20 °C (24h)	4 °C (24h)	25 °C (24h)	37 °C * (1h)	42 °C (1h)	60 °C (1h)	95 °C (5min)
**vB_Sen-TO17**	86.26 ± 2.6	85.71 ± 2.25	85.70 ± 3.47	71.42 ± 4.66	100.00	61.94 ± 3.16	66.78 ± 2.8	<0.01
**vB_Sen-E22**	62.46 ± 4.8	55.56 ± 6.54	100.00 ± 2.81	100.00 ± 5.18	100.00	100.00 ± 2.75	88.88 ± 3.16	<0.01

Values obtained at 37 °C (marked as *) were assessed as 100% and other values reflect these controls.

**Table 6:** Efficacy of phage plating of phages vB_Sen-TO17 and vB_Sen-E22 at different temperatures. Control temperature is marked with an asterisk.

Phage Name	Phage Propagation at Different Temperatures (Relative Phage Titer in %)			
	20 °C	25 °C	37 °C *	42 °C
**vB_Sen-TO17**	77.58 ± 2.66	83.78 ± 4.83	100.00	100.00 ± 6.21
**vB_Sen-E22**	100.00 ± 3.81	98.22 ± 4.77	100.00	100.00 ± 3.22

Values obtained at 37 °C (marked as *) were assessed as 100% and other values reflect these controls.

**Table 7:** Burst size of phages vB_Sen-TO17 and vB_Sen-E22 on different *S. enterica* strains at 42 °C. The burst size was calculated from samples untreated with chloroform during the one-step growth experiment. Mean values from three independent experiments with SD are presented.

Phage Name	*Salmonella enterica* Strain		Burst Size (PFU/cell)
	Serotype	No.	
vB_Sen-TO17	Typhimurium	12	58.88 ± 4.71
		13	79.17 ± 3.77
	Enteritidis	64	9.27 ± 2.57
		1392	-
vB_Sen-E22	Typhimurium	12	55.48 ± 4.82
		13	64.83 ± 6.36
	Enteritidis	64	18.43 ± 2.15
		1392	57.74 ± 8.47

## Supplementary Materials

The supplementary materials are available online at https://www.mdpi.com/1422-0067/21/22/8821/s1.
