# Supplementary material for: Bacteriophages vB_Sen-TO17 and vB_Sen-E22, Newly Isolated Viruses from Chicken Feces, Specific for Several Salmonella enterica Strains
Source: Int J Mol Sci. 2020 Nov 21;21(22):8821. doi: 10.3390/ijms21228821 (PMC7700153; doi:10.3390/ijms21228821)
Supplement: Supplementary file 1 [file ijms-21-08821-s001.zip › Sen-TO17-Supplementaty Tables 1 and 2.pdf]

Table S1. Genome annotations of phage vB_Sen-TO17

Locus tag	Start	Stop	Strand	Product
vBSenTO17_01	41290	339	-	hypothetical protein
vBSenTO17_02	662	423	-	putative spannin
vBSenTO17_03	1095	655	-	hypothetical protein
vBSenTO17_04	1427	1092	-	hypothetical protein
vBSenTO17_05	1401	2255	+	hypothetical protein
vBSenTO17_06	2743	2282	-	fibritin protein
vBSenTO17_07	3788	2745	-	putative head protein
vBSenTO17_08	3887	3789	-	hypothetical protein
vBSenTO17_09	5401	3908	-	Structural protein
vBSenTO17_10	6641	5418	-	terminase large subunit
vBSenTO17_11	7170	6664	-	hypothetical protein
vBSenTO17_12	7402	7304	-	hypothetical protein
vBSenTO17_13	7617	7399	-	putative NinH-like protein
vBSenTO17_14	7862	7614	-	Nin protein
vBSenTO17_15	8028	7855	-	hypothetical protein
vBSenTO17_16	8212	8021	-	hypothetical protein
vBSenTO17_17	8349	8209	-	hypothetical protein
vBSenTO17_18	8543	8346	-	hypothetical protein
vBSenTO17_19	9176	8694	-	Lysin / lysozyme
vBSenTO17_20	9456	9157	-	putative holin-like class I
vBSenTO17_21	9739	9458	-	putative holin
vBSenTO17_22	10213	9812	-	hypothetical protein
vBSenTO17_23	10548	10210	-	hypothetical protein
vBSenTO17_24	10790	10581	-	hypothetical protein
vBSenTO17_25	10987	10793	-	hypothetical protein
vBSenTO17_26	11616	10984	-	hypothetical protein
vBSenTO17_27	11783	11616	-	hypothetical protein
vBSenTO17_28	12025	11840	-	hypothetical protein
vBSenTO17_29	12186	12013	-	hypothetical protein
vBSenTO17_30	12343	12191	-	hypothetical protein
vBSenTO17_31	12603	12418	-	hypothetical protein
vBSenTO17_32	12775	12650	-	hypothetical protein
vBSenTO17_33	12943	12803	-	hypothetical protein
vBSenTO17_34	15263	12966	-	putative phage replicative helicase/primease
vBSenTO17_35	15582	15397	-	hypothetical protein
vBSenTO17_36	15800	15579	-	DNA-binding protein
vBSenTO17_37	15978	15868	-	hypothetical protein
vBSenTO17_38	15953	16627	+	hypothetical protein
vBSenTO17_39	16680	17162	+	hypothetical protein
vBSenTO17_40	17167	17442	+	hypothetical protein
vBSenTO17_41	17491	17649	+	hypothetical protein
vBSenTO17_42	17646	17888	+	hypothetical protein
vBSenTO17_43	17885	18046	+	hypothetical protein
vBSenTO17_44	18196	19479	+	hypothetical protein
vBSenTO17_45	19574	20269	+	hypothetical protein
vBSenTO17_46	20329	22590	+	DNA polymerase I

vBSenTO17_47	23003	22605	-	hypothetical protein
vBSenTO17_48	23109	22957	-	hypothetical protein
vBSenTO17_49	23002	23334	+	hypothetical protein
vBSenTO17_50	23325	23468	+	hypothetical protein
vBSenTO17_51	23459	23638	+	hypothetical protein
vBSenTO17_52	23635	24126	+	hypothetical protein
vBSenTO17_53	24123	25568	+	DNA helicase
vBSenTO17_54	25565	26062	+	HNH homing endonuclease
vBSenTO17_55	26062	26235	+	hypothetical protein
vBSenTO17_56	26236	26325	+	hypothetical protein
vBSenTO17_57	28408	26354	-	tailspike
vBSenTO17_58	30907	28421	-	putative tail protein
vBSenTO17_59	31335	30970	-	hypothetical protein
vBSenTO17_60	31847	31332	-	hypothetical protein
vBSenTO17_61	32344	31844	-	hypothetical protein
vBSenTO17_62	34575	32347	-	tail tape measure
vBSenTO17_63	34608	34820	+	hypothetical protein
vBSenTO17_64	35403	34930	-	hypothetical protein
vBSenTO17_65	35513	35797	+	hypothetical protein
vBSenTO17_66	36985	35819	-	putative tail protein
vBSenTO17_67	37401	36988	-	putative structural protein
vBSenTO17_68	37805	37401	-	putative tail protein
vBSenTO17_69	38161	37802	-	putative tail protein
vBSenTO17_70	38775	38161	-	Putative neck protein
vBSenTO17_71	39287	38778	-	putative head-tail joining protein
vBSenTO17_72	39485	39291	-	hypothetical protein
vBSenTO17_73	39869	39519	-	putative tail protein
vBSenTO17_74	40176	39886	-	putative head fiber protein
vBSenTO17_75	41287	40238	-	major capsid protein

Table S2. Genome annotations of phage vB_Sen-E22

Locus tag	Start	Stop	Strand	Product
vBSenE22_001	18	302	+	hypothetical protein
vBSenE22_002	299	748	+	swarming motility protein
vBSenE22_003	824	1339	+	hypothetical protein
vBSenE22_004	1405	1620	+	tail length tape-measure protein
vBSenE22_005	1662	1883	+	hypothetical protein
vBSenE22_006	1912	2613	+	putative metallopeptidase
vBSenE22_007	2684	2866	+	hypothetical protein
vBSenE22_008	2920	3558	+	tail fiber protein
vBSenE22_009	4001	4318	+	hypothetical protein
vBSenE22_010	4324	4773	+	spore cortex-lytic enzyme precursor
vBSenE22_011	4842	5012	+	hypothetical protein
vBSenE22_012	5012	5455	+	recombination related exonuclease
vBSenE22_013	6399	7346	+	hypothetical protein
vBSenE22_014	8087	7671	-	hypothetical protein
vBSenE22_015	9101	8109	-	hypothetical protein
vBSenE22_016	9368	9886	+	hypothetical protein
vBSenE22_017	10099	10287	+	hypothetical protein
vBSenE22_018	10387	10554	+	hypothetical protein
vBSenE22_019	10547	10753	+	hypothetical protein
vBSenE22_020	10844	11119	+	hypothetical protein
vBSenE22_021	11666	11938	+	hypothetical protein
vBSenE22_022	11989	12213	+	hypothetical protein
vBSenE22_023	12210	12401	+	hypothetical protein
vBSenE22_024	12801	12986	+	hypothetical protein
vBSenE22_025	13099	13446	+	hypothetical protein
vBSenE22_026	13900	14097	+	hypothetical protein
vBSenE22_027	14287	14454	+	hypothetical protein
vBSenE22_028	15296	15478	+	hypothetical protein
vBSenE22_029	15950	16150	+	hypothetical protein
vBSenE22_030	16417	16638	+	hypothetical protein
vBSenE22_031	16631	16795	+	hypothetical protein
vBSenE22_032	16905	17072	+	hypothetical protein
vBSenE22_033	17140	17433	+	hypothetical protein
vBSenE22_034	17433	17639	+	hypothetical protein
vBSenE22_035	17927	18121	+	hypothetical protein
vBSenE22_036	18164	18532	+	pyruvate formate-lyase
vBSenE22_037	18614	18919	+	hypothetical protein
vBSenE22_038	19041	19388	+	hypothetical protein
vBSenE22_039	19465	19746	+	hypothetical protein
vBSenE22_040	19739	20038	+	hypothetical protein
vBSenE22_041	20031	20450	+	hypothetical protein
vBSenE22_042	20404	20700	+	hypothetical protein
vBSenE22_043	20697	20981	+	hypothetical protein
vBSenE22_044	21092	21259	+	hypothetical protein
vBSenE22_045	21265	21435	+	hypothetical protein
vBSenE22_046	21590	22288	+	hypothetical protein
vBSenE22_047	22245	22694	+	hypothetical protein

vBSenE22_048	22978	23730	+	deoxynucleoside-5'-monophosphate kinase
vBSenE22_049	23743	24342	+	ATP-dependent Clp protease proteolytic subunit
vBSenE22_050	24499	25155	+	putative holin
vBSenE22_051	25152	25565	+	endolysin
vBSenE22_052	25643	26059	+	hypothetical protein
vBSenE22_053	26135	26566	+	hypothetical protein
vBSenE22_054	26559	26849	+	putative thioredoxin
vBSenE22_055	26947	27192	+	hypothetical protein
vBSenE22_056	27195	28058	+	serine/threonine protein phosphatase
vBSenE22_057	28058	28579	+	putative serine/threonine protein phosphatase
vBSenE22_058	28572	28694	+	hypothetical protein
vBSenE22_059	28747	29178	+	hypothetical protein
vBSenE22_060	29257	29508	+	hypothetical protein
vBSenE22_061	29565	29672	+	hypothetical protein
vBSenE22_062	29672	29953	+	hypothetical protein
vBSenE22_063	29950	30195	+	hypothetical protein
vBSenE22_064	30185	30511	+	hypothetical protein
vBSenE22_065	30508	30654	+	hypothetical protein
vBSenE22_066	30611	30811	+	hypothetical protein
vBSenE22_067	30808	31269	+	hypothetical protein
vBSenE22_068	31217	31588	+	capsid and scaffold A
vBSenE22_069	31643	31846	+	hypothetical protein
vBSenE22_070	31806	32294	+	hypothetical protein
vBSenE22_071	32287	32577	+	hypothetical protein
vBSenE22_072	32565	32798	+	hypothetical protein
vBSenE22_073	32798	32983	+	hypothetical protein
vBSenE22_074	32983	33591	+	hypothetical protein
vBSenE22_075	33653	34042	+	hypothetical protein
vBSenE22_076	34045	34761	+	hypothetical protein
vBSenE22_077	34748	35104	+	hypothetical protein
vBSenE22_078	37249	36905	-	hypothetical protein
vBSenE22_079	37376	37236	-	hypothetical protein
vBSenE22_080	37585	37373	-	hypothetical protein
vBSenE22_081	37737	37588	-	hypothetical protein
vBSenE22_082	38017	37787	-	hypothetical protein
vBSenE22_083	39122	38136	-	hypothetical protein
vBSenE22_084	39710	40231	+	hypothetical protein
vBSenE22_085	40725	40928	+	hypothetical protein
vBSenE22_086	41161	41412	+	hypothetical protein
vBSenE22_087	41513	41929	+	A2 protein
vBSenE22_088	41987	42184	+	putative A1 protein precursor
vBSenE22_089	42225	43946	+	A1 protein
vBSenE22_090	44012	44404	+	hypothetical protein
vBSenE22_091	44485	45219	+	deoxynucleoside-5'-monophosphatase
vBSenE22_092	45616	45371	-	hypothetical protein
vBSenE22_093	45720	45616	-	hypothetical protein
vBSenE22_094	45848	45720	-	hypothetical protein
vBSenE22_095	46276	46010	-	receptor-blocking protein

vBSenE22_096	46362	48149	+	receptor-binding protein
vBSenE22_097	48160	48642	+	putative SciB protein
vBSenE22_098	48642	49958	+	terminase large subunit
vBSenE22_099	50073	50510	+	nicking endonuclease
vBSenE22_100	50510	51727	+	portal protein
vBSenE22_101	51724	52218	+	tail fibers protein
vBSenE22_102	52222	52854	+	putative prohead protease
vBSenE22_103	52872	54248	+	major head protein precursor
vBSenE22_104	54308	54820	+	hypothetical protein
vBSenE22_105	54820	55587	+	tail completion protein
vBSenE22_106	55591	56076	+	tail tube terminator protein
vBSenE22_107	56103	57512	+	major tail protein
vBSenE22_108	57517	58416	+	tail fibers protein
vBSenE22_109	58413	58817	+	hypothetical protein
vBSenE22_110	58879	59247	+	hypothetical protein
vBSenE22_111	59330	63010	+	pore-forming tail tip protein
vBSenE22_112	63120	63734	+	distal tail protein
vBSenE22_113	63731	66580	+	tail protein Pb3
vBSenE22_114	66580	68637	+	tail protein Pb4
vBSenE22_115	68642	69064	+	putative tail protein
vBSenE22_116	69064	72414	+	chaperone of endosialidase
vBSenE22_117	72458	74545	+	tail fibers protein
vBSenE22_118	74849	74583	-	hypothetical protein
vBSenE22_119	75258	74812	-	putative deoxyUTP pyrophosphatase
vBSenE22_120	76130	75255	-	flap endonuclease
vBSenE22_121	76612	76130	-	D14 protein
vBSenE22_122	78454	76616	-	putative exonuclease subunit 2
vBSenE22_123	79412	78435	-	putative recombination endonuclease subunit D12
vBSenE22_124	80222	79449	-	D11 protein
vBSenE22_125	80499	80215	-	hypothetical protein
vBSenE22_126	82072	80720	-	putative ATP-dependent helicase
vBSenE22_127	82566	82069	-	hypothetical protein
vBSenE22_128	85126	82559	-	DNA polymerase
vBSenE22_129	86079	85189	-	putative DNA replication primase
vBSenE22_130	87599	86076	-	putative replicative DNA helicase
vBSenE22_131	88398	87631	-	D5 protein
vBSenE22_132	89161	88391	-	NAD-dependent DNA ligase subunit B
vBSenE22_133	90335	89364	-	NAD-dependent DNA ligase subunit A
vBSenE22_134	90516	90328	-	hypothetical protein
vBSenE22_135	90910	90602	-	transcriptional coactivator p15
vBSenE22_136	91257	90961	-	hypothetical protein
vBSenE22_137	91704	91294	-	D3 protein
vBSenE22_138	92041	91811	-	hypothetical protein
vBSenE22_139	92738	92034	-	D2 protein
vBSenE22_140	93040	92807	-	hypothetical protein
vBSenE22_141	95813	93024	-	putative replication origin binding protein
vBSenE22_142	96825	96436	-	tail tube protein
vBSenE22_143	97263	96835	-	tail tube protein
vBSenE22_144	97778	97266	-	hypothetical protein

vBSenE22_145	98574	97759	-	NAD-dependent protein deacetylase of SIR2 family
vBSenE22_146	98702	98574	-	hypothetical protein
vBSenE22_147	98874	98671	-	hypothetical protein
vBSenE22_148	99165	98884	-	hypothetical protein
vBSenE22_149	101139	99265	-	anaerobic ribonucleoside-triphosphate reductase
vBSenE22_150	101492	102244	+	phosphate starvation-inducible protein
vBSenE22_151	102246	102467	+	hypothetical protein
vBSenE22_152	102505	104943	+	ribonucleotide reductase of class Ia (aerobic)
vBSenE22_153	104999	105481	+	putative H-N-H-endonuclease
vBSenE22_154	105548	106693	+	putative aerobic ribonucleoside diphosphate
vBSenE22_155	106690	107223	+	putative dihydrofolate reductase
vBSenE22_156	107223	108077	+	thymidylate synthase
vBSenE22_157	108171	108452	+	hypothetical protein
vBSenE22_158	108452	108928	+	ribonuclease H
